# Non-Small-Cell Lung Cancer Clinicopathologic Features and Survival Outcomes in Asian Pacific Islanders Residing in the United States: A SEER Analysis

**DOI:** 10.1155/2015/269304

**Published:** 2015-01-15

**Authors:** Muhammad Saad Hamid, Raji Shameem, Khalid Gafoor, Jason George, Bushra Mina, Kevin Sullivan

**Affiliations:** ^1^Department of Internal Medicine, Detroit Medical Center/Wayne State University, Detroit, MI 48201, USA; ^2^Department of Hematology-Oncology, Fox Chase Cancer Center, Philadelphia, PA 19111, USA; ^3^Department of Internal Medicine, Lenox Hill Hospital, New York, NY 10065, USA; ^4^Department of Pulmonology, Lenox Hill Hospital, New York, NY 10065, USA; ^5^Department of Hematology-Oncology, Lenox Hill Hospital, New York, NY 10065, USA

## Abstract

*Background*. The objective of our study was to ascertain racial/ethnic disparities in Asian/Pacific Islanders (API) for non-small-cell lung cancer (NSCLC) clinicopathologic features and survival outcomes based on various tumor characteristics and treatment modalities. *Method*. SEER database identified invasive NSCLC cases from 2004 to 2010. Variables included American Joint Committee on Cancer (AJCC) stage 7, tumor grade, tumor size, histology, age, marital status, radiation, surgery, and reason for no surgery. The Kruskall-Wallis test and the *Z* test were used to examine differences between races/ethnicities and the referent, non-Hispanic white (NHW). Multivariate Cox proportional analyses were used to establish the weight of the prognostic significance contributing to disease-specific survival (DSS) in each AJCC stage. *Result*. Improved DSS was seen in API across stage I (HR: 0.78), stage II (HR: 0.79), and stage IV (HR: 0.86), respectively, compared to the referent NHW (*P* < 0.01). Prognosis was improved by being married, being female gender, AIS histology, and birth outside the US (*P* < 0.01). *Conclusion*. We have demonstrated improved survival among API in early stage and stage IV NSCLC. Further research is necessary to clarify the role of lifestyle and tumor biology for these differences.

## 1. Background

**Figure 1:**
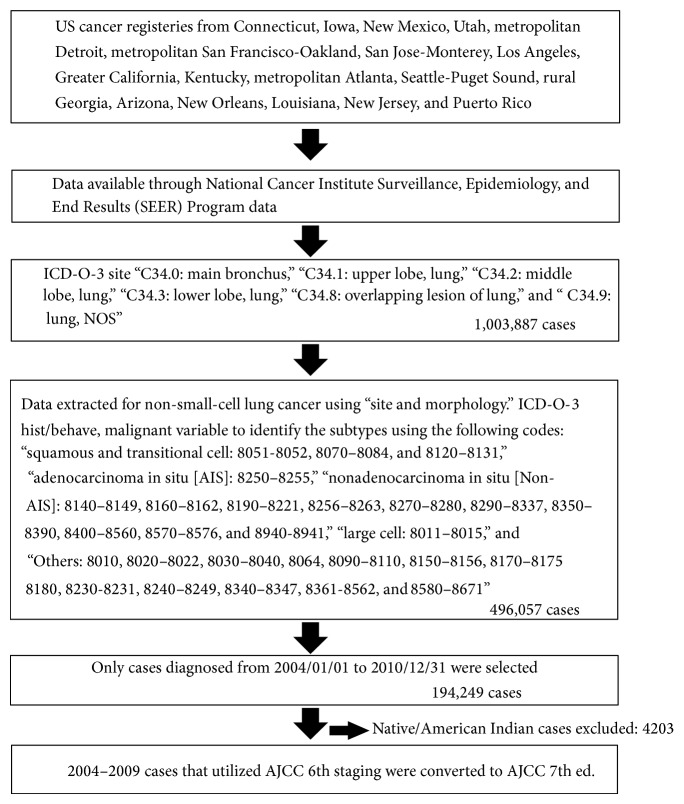
Selection of the non-small-cell lung cancer cases included in the study.

Lung cancer is the second most common cancer in both men and women with an estimated 224,210 cases expected to be diagnosed in 2014 in the United States (US) [1]. It is also the leading cause of cancer related deaths in the US accounting for 27% of all cancer related deaths [1]. The majority of lung cancer cases fall under the category of non-small-cell lung cancer (NSCLC) [1].  Figure 1 shows the selection of the non-small-cell lung cancer cases included in the study.

Racial/ethnic disparities have been shown to influence survival outcomes in NSCLC [2–4]. Disparities in survival outcomes among racial/ethnic groups may be attributed to a complex interaction between genetic and lifestyle factors [4, 5]. Compared to non-Hispanic whites (NHW), Blacks have a higher incidence of lung cancer and more advanced disease at diagnosis with worse survival outcomes [6–8]. Despite a lower incidence, Hispanics are more likely to be diagnosed with advanced disease with poor outcomes compared to NHW [2, 9]. Asian/Pacific Islanders (API) have a lower incidence of NSCLC compared to NHW [10]. Interestingly, previous literature has shown that cancer related mortality is favorable in API compared to other racial/ethnic groups for early stage (stages IA and IB) NSCLC [11, 12]. However, survival outcomes in API and other racial/ethnic groups based on the recently published American Joint Committee on Cancer (AJCC) 7th edition have not been evaluated in detail [13].

In this study, our primary objective was to utilize an established large nationwide cancer registry to ascertain racial/ethnic disparities in NSCLC clinicopathologic features and survival outcomes.

## 2. Methods

We used the National Cancer Institute (NCI) Surveillance, Epidemiology, and End Result (SEER) cancer registry that collects large observational data across 18 cancer registry sites. The database was accessed using the SEER^*^Stat 8.1.5, http://seer.cancer.gov/seerstat, assessed May 01, 2014. To be eligible, we identified patients diagnosed between 01/01/2004 to 31/12/2010 with NSCLC (ICD-O-3 Site C34.0–C34.9) based on the selected histology codes: squamous and transitional cell: 8051-8052, 8070–8084, and 8120–8131, adenocarcinoma in situ [AIS]: 8250–8255, nonadenocarcinoma in situ [Non-AIS]: 8050, 8140–8149, 8160–8162, 8190–8221, 8256–8263, 8270–8280, 8290–8337, 8350–8390, 8400–8560, 8570–8576, and 8940-8941, large cell: 8011–8015, and “Others”: 8010, 8020–8022, 8030–8040, 8046, 8090–8110, 8150–8156, 8170–8175, 8180, 8230-8231, 8240–8249, 8340–8347, 8561-8562, and 8580–8671. We utilized the time period stated, due to the ability to restage the tumors to the latest AJCC 7th edition using data from the collaborative stage variables. To investigate any existing treatment or tumor racial/ethnic disparities and disease-specific survival (DSS), racial/ethnic groups were categorized as NHW, Hispanics, Blacks, and API. Clinicopathologic characteristics included age at diagnosis, gender, birth country, marital status, tumor grade, tumor size, AJCC stage, and histology. Treatment variables included radiation, surgery, and radiation/surgery sequence. Decade long time intervals “<30 years,” “30–39,” “40–49,” “50–59,” “60–69,” “70–79,” and “>80 years” were used to categorize age. Marital status was described using “Single,” “Married,” or “Others,” a term that is inclusive of divorced, widowed, or separated individuals. Birth within the US or outside was used to monitor “the immigration effect.” The variable “Tumor size” reflected the size of the tumor mass and was classified categorically to “<30 mm”, “30–50 mm”, “50–70 mm”, “>70 mm” or in cases with no recorded tumor mass to “No mass was found”. All forms of radiation were collectively grouped as “Radiation received,” while all forms of cancer directed surgeries were coded collectively as “Surgery performed.” Cases after 2010 were excluded to allow a minimum of 12 months of follow-up period.

### 2.1. Statistical Analysis

The Kruskall-Wallis nonparametric test was employed to examine the differences that may exist among various racial/ethnic groups and tumor characteristics. The difference between the racial/ethnic groups and the reasons for no surgery was measured using Fisher's exact test. The end point was DSS which was measured in months from the date of diagnosis to death due to lung cancer or censoring, which included either being alive, lost to follow-up, or died due to other causes.

Multivariate Cox proportional hazard models were used to establish the weight of different characteristics (grade, age at diagnoses, tumor size, histology, marital status, race/ethnicity, gender, radiation, surgery, and radiation/surgery sequence) on prognostic significance contributing to the survival in each respective AJCC stage. The *Z* test was employed to examine the proportional differences that may exist between the referent NHW and other race/ethnic groups. The models were constructed using IBM SPSS Statistical software, version 21.0 (IBM Corp. Released 2012, IBM SPSS Statistics for Windows, Version 21.0, Armonk, NY: IBM Corp.).

## 3. Results

Our database yielded 190,046 patients with NSCLC: 145646 (76.6%) NHW, 10350 (5.4%) Hispanics, 22525 (11.9%) Blacks, and 11525 (6.1%) API (Table 1).

### 3.1. Stage

Compared to NHW stage I diagnosis (17.8%), Blacks had the least proportion (12.4%) preceded by Hispanics (13.5%) and API (14.3%) (*P* < 0.05). NHW had the most stage II diagnosis (13.1%), followed by Blacks (12.7%), API (11.7%), and Hispanics (11.4%) (*P* < 0.05). Blacks had the highest stage III diagnoses (20.5%), followed by the referent NHW (18.5%), Hispanics (17.7%), and API (17.2%) (*P* < 0.05). API had the highest stage IV diagnoses (49.3%), followed by Hispanics (47.7%), Blacks (47.4%), and the referent NHW (42.6%), respectively.

### 3.2. Grade

Compared to the referent NHW's Grades 1 (5.0%) and 2 (16.5%) statuses, Blacks had the least amount of Grade 1 (3.3%) and Grade 2 (14.8%) tumors, while Hispanics (5.4%) and API (5.7%) both had relatively greater Grade 1 representations, respectively (*P* < 0.05). Regarding high grade tumors, API had significantly the lowest proportions of both Grade 3 (25.7%) and Grade 4 (1.8%) cases, compared to NHW. Hispanics also had lower Grade 3 (26.8%) diagnoses, compared to NHW, while Blacks had greater proportions (28.2%).

### 3.3. Histology

Squamous and transitional cell diagnoses were significantly less common in API (15.8%) and Hispanics (19.2%) compared to NHW (23.6%) (*P* < 0.05). Although Blacks had greater shares (24.1%), this was nonsignificant. Compared to NHWs' AIS (4.1%) and Non-AIS (38.3%) histological diagnoses, Hispanics had greater proportions (5.0% and 41.9%), with API having the greatest representation (6.3% and 49.6%). Alternatively, Blacks yielded the fewest AIS (2.8%) cases in our study (*P* < 0.05). For large cell carcinoma in the referent group (3.4%), both API (2.5%) and Hispanics (3.0%) ranked lower, while a greater share was found among Blacks (4.1%) (*P* < 0.05).

### 3.4. Age

Compared to NHW's later mean age at diagnosis of 68.86 years ± 11.239, an earlier onset was observed among API (68.05 ± 12.315 years), Hispanics (67.40 ± 12.395 years), and Blacks (64.65 ± 11.467 years) (*P* < 0.05). Greater than 50% of cases among Blacks were seen in the 5th (25.4%) and 6th (31.3%) decades, respectively, compared to the majority of the cases that presented later in the 6th and 7th decades among other ethnicities (*P* < 0.05).

### 3.5. Marital Status

In our study, Blacks (29.2%) had the highest “single” status, followed by Hispanics (15.4%), and NHW (10.5%), with lowest observations noted among API (9.5%). Blacks (32.9%) and NHW (33.0%) had higher “Others” status, with relative lower proportions observed amongst Hispanics (29.3%) and API (22.5%) (*P* < 0.05). Finally, married individuals were significantly more common among AIP (64.9%) and less common among Blacks (33.6%) compared to NHW (33.0%).

### 3.6. Birth Country

Significant majority of the API (58.2%) were born outside the United States (US). A greater proportion of Hispanics (34.9%), compared to NHW (4.1%), and Blacks (1.5%) are born outside (*P* < 0.05).

### 3.7. Tumor Size

“No tumor was found” in 0.3% of the Black population, compared to (0.4%) NHW (*P* < 0.05). Both NHW (31.0%) and API (27.8%) had the greater proportion of tumor ≤30 mm, compared to Blacks (25.1%) and Hispanics (26.7), respectively. A similar trend of proportionality was observed in tumors greater than 30 mm but not more than 50 mm, with API (23.7%) and NHW (22.6%) being higher, compared to Blacks (22.0%) and Hispanics (21.0%) (*P* < 0.05). Alternatively, Blacks had relatively greater proportion of tumors greater than 50 mm, followed by Hispanics, NHW, and API, respectively.

### 3.8. Radiation

With regard to stage I NSCLC cases, radiation was less frequently utilized in both API (11.0%) and Hispanics (12.2%) compared to NHW (16.3%) whereas greater proportions of Blacks (18.9%) were treated with radiation (*P* < 0.01). This trend was also observed in stage II cases, with relatively more NHW (33.9%) and Blacks (36.9%) than API (26.5%) and Hispanics (28.6%) receiving radiation as part of their treatment (*P* < 0.01). Higher rates of Blacks (57.3%) had radiation as part of the treatment in stage III followed by NHW (55.3%), API (50.5%), and Hispanics (48.2%) (*P* < 0.01). Similarly, Blacks (44.9%) had the highest proportions of radiation utilization compared to NHW (44.1%), API (41.0%), and Hispanics (39.1%) in the stage IV cohort (*P* < 0.01).

### 3.9. Surgery

For AJCC stage I cases, a greater proportion of API (80.4%) were treated with cancer directed surgery compared to Hispanics (75.4%), NHW (75.6%), and Blacks (65.7%) (*P* < 0.01). Blacks (35.1%) had the lowest rate of surgical treatment in stage II while NHW (47.3%) had the highest rate followed by API (44.7%) and Hispanics (44.7%) (*P* < 0.01). Compared to NHW (17.0%), lower rates of surgical treatment in Blacks (12.0%) were observed while API (20.4%) and Hispanics (19.4%) had greater proportions that underwent surgery (*P* < 0.01). NHW (4.5%) had the highest proportion of cancer directed surgery compared to Hispanics (3.8%), API (3.6%), and Blacks (3.5%) in stage IV NSCLC (*P* < 0.01).

### 3.10. Reason for No Surgery

The most common reason for no surgery for all ethnicities was because it was “not recommended.” This reason was proportionally more common among API and least among Blacks (*P* < 0.05) for all AJCC stages except stage I (*P* < 0.05). In contrast, API had the highest proportion of refusal for surgical treatment in early stage NSCLC patients (*P* < 0.05).

### 3.11. Survival Analyses

Multivariate Cox proportional models were utilized to analyze the variables contributing to the DSS among different AJCC stage.

### 3.12. Patient Demographics

Demographic variables that had improved survival at each AJCC stage were; female gender, and being married, (*P* < 0.05). Immigrants born outside the US had significant improved survival outcome in comparison to US born patients. Patients with stage II diagnosed at age 70–79 (hazard ratio [HR]: 4.077, *P* < 0.05) and >80 (HR: 5.14, *P* < 0.05) had poor outcomes; Patients >80 years had worsened survival among stage IV (HR: 1.626, *P* < 0.05). API had a significantly improved survival in stage I (HR: 0.775, *P* < 0.05), stage II (HR: 0.791, *P* < 0.05), and stage IV (HR: 0.858, *P* < 0.05). This improvement was not observed in stage III (HR: 0.966, *P* > 0.1). Unlike API, both Hispanics and Blacks did not have impact on the survival favorably compared to NHW (Table 3).

### 3.13. Clinicopathologic Features

Higher grade was uniformly associated with poor prognosis across all the stages (*P* < 0.05). However both AIS and Non-AIS diagnoses (with the exception of stage II, HR: 0.966, *P* > 0.05) were both associated with improved survival compared to the referent squamous and transitional diagnosis, with AIS being the more favorable diagnosis (Table 2).

### 3.14. Treatment Modality

Treatment with radiation was associated with favorable 5-year prognosis (stage I HR: 0.693; stage II HR: 0.623; stage III HR: 0.60, and stage IV: 0.917, *P* < 0.01). Surgical treatment favorably impacted stage I (HR: 0.231), and stage II (HR: 0.282) survival respectively, (*P* < 0.01).

## 4. Discussion

This study utilized the SEER database to examine racial/ethnic disparities in NSCLC clinicopathologic features and stage-based survival outcomes. API were more likely to be diagnosed with AIS histology but yet presented with late stage disease. Our analysis showed that cancer directed surgery and radiation therapy were significantly less likely to be offered to API compared to NHW. Despite this, compared to NHW, API had increased disease-specific survival for early stage (I and II) and stage IV NSCLC. This analysis determined that survival disparities are also seen in API based on the recent AJCC 7th edition staging system. Previous retrospective analyses have shown API to have decreased mortality compared to NHW for stage I disease, with an overall survival advantage regardless of smoking status which is consistent with our results [7–9]. Our analysis also found increased survival in API with stage II disease compared to NHW. Stage IV disease was seen more frequently in API than in NHW with lower rates of cancer directed surgery and radiation therapy. Despite this, there was a survival advantage for API compared to NHW in stage IV NSCLC which is consistent with prior studies [14].

Improved outcomes in API may be attributed to favorable demographic and clinicopathologic features demonstrated in our analysis including being married, birth outside of the US, AIS histology, and earlier age at diagnosis. Regarding treatment modalities in stage IV, despite improved survival, the API cohort was less likely to receive cancer directed surgery compared to NHW. Pertinently, there was greater proportion of surgery which was not part of the treatment plan. According to Chang et al., API as a group had better overall survival after NSCLC diagnosis compared to NHW, and single marital status was associated with decreased survival in the API population, which is consistent with our results [8].

NSCLC is a heterogeneous disease that is influenced by genetic, lifestyle, and socioeconomic elements. These elements are likely major factors in the disparate presentations and outcomes among different racial/ethnic groups. Smoking status is an important prognostic indicator, with an improvement in overall and disease-specific survival in never smokers compared to patients with a smoking history [9, 11]. Response to therapy including surgery, chemotherapy, and radiation is also improved in never smokers even in advanced disease [9]. Unlike NHW and black patients diagnosed with NSCLC, a relatively high percentage of never smokers are seen in the API US population [12]. However, besides smoking status, additional factors may account for improved outcomes because Asian ethnicity independently is a favorable prognostic indicator for overall survival in both smokers and never smokers [9]. Lower socioeconomic status (SES) is associated with increased lung cancer incidence [15]. In addition to a higher prevalence of smoking in lower SES groups, they are unlikely to receive adequate health care. In prior observational studies, Blacks were less likely to receive surgery, chemotherapy, or radiation for stage III disease and were less likely to receive chemotherapy for stage IV disease in comparison to NHW [16–18]. In our study, cancer directed surgery was less likely to be offered to Blacks compared to NHW. However, our study is unique in that it demonstrates that radiation is more significantly likely to be administered to Blacks diagnosed with NSCLC. Poor access to quality health care is a major factor in racial/ethnic disparities, which have shown that when equivalent health care access is provided, survival outcomes become comparable [3, 13, 19, 20]. Further research is necessary to determine whether lung cancer treatment is suboptimal in API residing in the US.

Overexpression of the epidermal growth factor receptor (EGFR) leading to aberrant tyrosine kinase mediated signaling is implicated in approximately 70% of NSCLC cases and is associated with a poor prognosis [21]; EGFR tyrosine kinase inhibitors (TKI) were developed as a potential therapeutic option to improve outcomes. A greater understanding of the activity of TKI has led to the discovery that the efficacy of these inhibitors is dependent on the presence of EGFR activating mutations instead of the degree of EGFR overexpression. EGFR activating mutations are seen more commonly in females, AIS histology, never or light smokers, and East Asians [22, 23]. The prevalence of EGFR activating mutations in other racial/ethnic groups such as Blacks and NHW appears to be highly variable [24–26]. Improved survival in API potentially could be due to the presence of these mutations; however, randomized controlled trials have not shown an overall survival benefit with TKI therapy in the adjuvant, stage III maintenance, first-line metastatic, and second-line treatment settings [27–31].

This study has several limitations. It is a retrospective analysis where data was collected by medical record review. This could have led to incorrect classification of race/ethnicity and tumor classification. In our analysis, we came across cases with insufficient data labeled “NOS.” However, we found the number of missing cases to be proportional among different racial/ethnic groups. In addition, we were not able to account for both genetic and lifestyle factors linked to NSCLC including testing for EGFR, KRAS, and ALK mutations, familial history, smoking history, and occupational exposure to carcinogens. We were also not able to determine specific chemotherapy regimens given to patients.

This is the first SEER analysis to utilize the recent AJCC 7th edition to determine survival outcomes in API compared to NHW. Improved survival outcomes were seen in API for both early and advanced stage disease. Interestingly, in all stages, except for stage III, there was a significant survival benefit. This may be due to an insufficient sample size but also may be due to disparities in tumor biology and lifestyle factors specific for this stage. Further research is necessary to gain a better understanding of the NSCLC outcomes in the API population residing in the US.

## Figures and Tables

**Table 1:** Baseline demographic and clinicopathologic of the study cohort.

Characteristics	NHW (145464)		Hispanic (10350)		Black (22525)		API (11525)	
	*f*	%	*f*	%	*f*	%	*f*	%
Grade (*P* < 0.01)								
Grade I	8027	5	632	5.4^*^	817	3.3^*^	724	5.7^*^
Grade II	26678	16.5	1849	15.7	3665	14.8^*^	2106	16.6
Grade III	44361	27.5	3158	26.8	6968	28.2	3250	25.7^*^
Grade IV	3590	2.2	258	2.2	554	2.2	233	1.8^*^
Unknown	78658	48.8	5871	49.9^*^	12710	51.4^*^	6355	50.2^*^
AJCC stage 7 (*P* < 0.01)								
Stage I	28649	17.8	1588	13.5^*^	3062	12.4^*^	1815	14.3
Stage II	21121	13.1	1345	11.4^*^	3150	12.7^*^	1481	11.7^*^
Stage IIIa	22119	13.7	1468	12.5^*^	3711	15.0^*^	1514	12.0^*^
Stage IIIb	4830	3	330	2.8	899	3.6^*^	469	3.7^*^
Stage IV	68745	42.6	5619	47.7^*^	11703	47.4^*^	6246	49.3^*^
Histology (*P* < 0.01)								
Squamous cell/transitional cell carcinoma	38005	23.6	2265	19.2^*^	5955	24.1	1999	15.8^*^
Adenocarcinoma in situ [AIS]	6678	4.1	587	5.0^*^	686	2.8^*^	794	6.3^*^
Nonadenocarcinoma in situ [non-AIS]	61860	38.3	4931	41.9^*^	9512	38.5	6282	49.6^*^
Large cell carcinoma	5483	3.4	356	3.0^*^	1013	4.1^*^	315	2.5^*^
Others	49288	30.6	3629	30.8^*^	7548	30.5	3278	25.9^*^
Age								
Mean ± standard deviation	68.86 ± 11.239		67.40 ± 12.395^*^		64.65 ± 11.467^*^		68.05 ± 12.315^*^	
Median (range)	70 (15–99)		69 (15–99)		65 (15–99)		69 (20–99)	
Age grouping (*P* < 0.01)								
<30	203	0.1	73	0.6^*^	41	0.2	34	0.3^*^
30–39	815	0.5	170	1.4^*^	192	0.8^*^	192	1.5^*^
40–49	7353	4.6	761	6.5^*^	2022	8.2^*^	707	5.6^*^
50–59	24812	15.4	1924	16.3^*^	6279	25.4^*^	2210	17.4^*^
60–69	47243	29.3	3253	27.6^*^	7734	31.3^*^	3278	25.9^*^
70–79	51282	31.8	3640	30.9	5806	23.5^*^	3910	30.9^*^
80+	29606	18.4	1947	16.5^*^	2640	10.7^*^	2337	18.4
Gender (*P* < 0.01)								
Male	85908	53.3	6407	54.4^*^	14086	57.0^*^	7241	57.2^*^
Female	75406	46.7	5361	45.6^*^	10628	43.0^*^	5427	42.8^*^
Birth country (*P* < 0.01)								
United States	108817	67.5	3816	32.4^*^	18836	76.2^*^	2190	17.3^*^
Outside the United States	6565	4.1	4106	34.9^*^	376	1.5^*^	7369	58.2^*^
Unknown	45932	28.5	3846	32.7^*^	5502	22.3^*^	3109	24.5^*^
Marital status (*P* < 0.01)								
Single	16835	10.4	1815	15.4^*^	7212	29.2^*^	1200	9.5^*^
Married	85907	53.3	6074	51.6	8299	33.6^*^	8216	64.9^*^
Others	53186	33	3452	29.3^*^	8136	32.9	2851	22.5^*^
Unknown	5386	3.3	427	3.6^*^	1067	4.3^*^	400	3.2
Tumor size (*P* < 0.01)								
No tumor found	713	0.4	55	0.5	74	0.3^*^	45	0.4
≤30 mm	49950	31	3147	26.7^*^	6205	25.1^*^	3528	27.8^*^
>30 mm and ≤50 mm	36401	22.6	2470	21.0^*^	5449	22.0^*^	3008	23.7^*^
>50 mm and ≤70 mm	19615	12.2	1461	12.4	3399	13.8^*^	1577	12.4
>70	14848	9.2	1160	9.9^*^	3060	12.4^*^	1122	8.9
Unknown	393787	24.7	3475	29.5^*^	6527	26.4^*^	3388	26.7^*^

^*^ *P* < 0.05 using *Z* test when c/w NHW.

Ca.: carcinoma; *f*: frequency; *P*: *P* value; %, percentage; mm: millimeter; API: Asian Pacific Islanders; NHW: non-Hispanic whites.

**Table 2:** Baseline treatment characteristics of the racial/ethnic racial cohorts among the AJCC stages.

	NHW (28649)		Hispanics (1588)		Blacks (3062)		API (1815)	
	*f*	%	*f*	%	*f*	%	*f*	%
AJCC stage I								
Radiation (*P* < 0.01)								
Radiation not received	23652	82.6	1385	87.2^*^	2450	80.8^*^	1601	88.2^*^
Radiation received	4666	16.3	194	12.2^*^	578	18.9^*^	200	11
Unknown	331	1.2	9	0.6^*^	34	1.1	14	0.8
Cancer directed surgery (*P* < 0.01)								
Not performed	6961	24.3	389	24.5	1033	33.7^*^	354	19.5^*^
Performed	21582	75.6	1197	75.4	2012	65.7^*^	1459	80.4^*^
Reason for no surgery (*P* < 0.05)^a^								
Died	21	0.3	1	0.3	3	0.3	0	0
Not recommended	6014	86.3	328	84.3	873	84.5	294	83.1
Patient refusal	450	6.4	28	7.1	81	7.8	41	11.6^*^
Unknown	582	8.4	34	8.7	93	9	21	5.9

	NHW (21121)		Hispanics (1345)		Blacks (3150)		API (1481)	
	*f*	%	*f*	%	*f*	%	*f*	%

AJCC stage II								
Radiation (*P* < 0.01)								
Radiation not received	13590	64.3	941	70.0^*^	1954	62.0^*^	1075	72.6^*^
Radiation received	7159	33.9	384	28.6^*^	1144	36.3^*^	392	26.5^*^
Unknown	372	1.8	20	1.5	52	1.7	14	0.9^*^
Cancer directed surgery (*P* < 0.01)								
Not performed	11083	52.7	743	55.3	2038	64.9^*^	818	55.3^*^
Performed	9937	47.3	601	44.7	1101	35.1^*^	661	44.7^*^
Reason for no surgery (*P* < 0.05)^a^								
Died	37	0.3	5	0.7	8	0.4	2	0.2
Not recommended	10089	91	672	90.4	1826	89.6^*^	760	92.9^*^
Patient refusal	308	3.1	21	2.8	56	2.7	28	3.4
Unknown	750	6.7	46	6.1	159	7.8	30	3.7^*^

	NHW (26949)		Hispanics (1798)		Blacks (4610)		API (1983)	
	*f*	%	*f*	%	*f*	%	*f*	%

AJCC stage III								
Radiation (*P* < 0.01)								
Radiation not received	11542	42.8	908	50.5^*^	1885	40.9^*^	955	48.2^*^
Radiation received	14909	55.3	867	48.2^*^	2641	57.3^*^	1002	50.5^*^
Unknown	498	1.8	23	1.3	84	1.8	26	1.3^*^
Cancer directed surgery (*P* < 0.01)								
Not performed	22228	83	1448	80.6^*^	4037	88.0^*^	1577	79.6^*^
Performed	4562	17	349	19.4^*^	548	12.0^*^	403	20.4^*^
Reason for no surgery (*P* < 0.01)^a^								
Died	52	0.2	1	0	9	0.2	2	0.1
Not recommended	20685	93.1	1353	93.4	3713	92.0^*^	1504	95.4^*^
Patient refusal	353	1.6	20	1.4	53	1.3	21	1.3
Unknown	1297	5.8	75	5.2	287	7.1^*^	53	3.7^*^

	NHW (68745)		Hispanics (5619)		Blacks (11703)		API (6246)	
	*f*	%	*f*	%	*f*	%	*f*	%

AJCC stage IV								
Radiation (*P* < 0.01)								
Radiation not received	37450	54.5	3356	59.7^*^	6285	53.7	3628	58.1^*^
Radiation received	30306	44.1	2195	39.1^*^	5259	44.9	2562	41.0^*^
Unknown	989	1.4	68	1.2	159	1.4	56	0.9^*^
Cancer directed surgery (*P* < 0.01)								
Not performed	65265	95.5	5399	96.2^*^	11233	96.5^*^	6014	96.4^*^
Performed	3107	4.5	213	3.8^*^	410	3.5^*^	222	3.6^*^
Reason for no surgery (*P* < 0.01)^a^								
Died	141	0.2	14	0.3	28	0.3	14	0.2
Not recommended	61220	93.8	5112	94.7^*^	10331	92^*^	5796	96.3^*^
Patient refusal	766	1.2	41	0.8^*^	138	1.2	58	0.9
Unknown	3511	5.4	239	4.4^*^	796	7.1^*^	156	2.6^*^

^*^ *P* < 0.05 using *Z* test when c/w NHW; ^a^Fisher's exact test was used to test difference among the races.

*f*: frequency; *P*: *P* value; %, percentage; API: Asian Pacific Islanders; NHW: non-Hispanic whites.

**Table 3:** Multivariate Cox proportional analysis used to ascertain the contributions of the demographic, clinicopathologic, and treatment features to the DSS among the different AJCC stages.

Characteristics	Stage I		Stage II		Stage IIIa		Stage IIIb		Stage IV	
	HR	*P*	HR	*P*	HR	*P*	HR	*P*	HR	*P*
Grade										
Grade I	Referent		Referent		Referent		Referent		Referent	
Grade II	1.589	*P* < 0.01	1.15	*P* < 0.01	1.049	*P* > 0.05	1.482	*P* < 0.01	1.24	*P* < 0.01
Grade III	1.836	*P* < 0.01	1.348	*P* < 0.01	1.208	*P* < 0.01	1.507	*P* < 0.01	1.467	*P* < 0.01
Grade IV	1.884	*P* < 0.01	1.426	*P* < 0.01	1.346	*P* < 0.01	1.27	*P* > 0.05	1.525	*P* < 0.01
Histology										
Squamous cell/transitional cell Ca.	Referent		Referent		Referent		Referent		Referent	
Adenocarcinoma in situ [AIS]	0.642	*P* < 0.01	0.739	*P* < 0.01	0.623	*P* < 0.01	0.539	*P* < 0.05	0.739	*P* < 0.01
Nonadenocarcinoma in situ	0.928	*P* < 0.05	0.966	*P* > 0.05	0.887	*P* < 0.01	0.956	*P* > 0.05	0.935	*P* < 0.01
Large cell carcinoma	1.088	*P* > 0.05	1.079	*P* > 0.05	0.927	*P* > 0.05	1.049	*P* > 0.05	1.05	*P* > 0.05
Others	0.896	*P* < 0.05	0.983	*P* > 0.05	0.906	*P* < 0.01	0.952	*P* > 0.05	1.05	*P* < 0.05
Age grouping										
<30	Referent		Referent		Referent		Referent		Referent	
30–39	1.252	*P* > 0.05	2.795	*P* > 0.05	1.332	*P* > 0.05	0.879	*P* > 0.05	1.04	*P* > 0.05
40–49	1.992	*P* > 0.05	3.091	*P* > 0.05	1.477	*P* > 0.05	1.298	*P* > 0.05	1.111	*P* > 0.05
50–59	2.091	*P* > 0.05	3.257	*P* > 0.05	1.426	*P* > 0.05	1.354	*P* > 0.05	1.215	*P* > 0.05
60–69	2.28	*P* > 0.05	3.455	*P* > 0.05	1.535	*P* > 0.05	1.338	*P* > 0.05	1.28	*P* > 0.05
70–79	2.639	*P* > 0.05	4.077	*P* < 0.05	1.726	*P* > 0.05	1.486	*P* > 0.05	1.409	*P* > 0.05
80+	3.025	*P* > 0.05	5.14	*P* < 0.05	2.083	*P* > 0.05	1.795	*P* > 0.05	1.626	*P* < 0.05
Gender										
Male	Referent		Referent		Referent		Referent		Referent	
Female	0.833	*P* < 0.01	0.831	*P* < 0.01	0.845	*P* < 0.01	0.8	*P* < 0.01	0.854	*P* < 0.01
Birth country										
United States	Referent		Referent		Referent		Referent		Referent	
Outside the United States	0.86	*P* < 0.01	0.915	*P* > 0.05	0.8	*P* < 0.01	0.825	*P* < 0.05	0.875	*P* < 0.01
Marital status										
Single	Referent		Referent		Referent		Referent		Referent	
Married	0.874	*P* < 0.01	0.915	*P* < 0.05	0.887	*P* < 0.01	0.771	*P* < 0.01	0.842	*P* < 0.01
Others	1.012	*P* > 0.05	1.001	*P* > 0.05	0.969	*P* > 0.05	0.922	*P* > 0.05	0.968	*P* > 0.05
Races										
Non-Hispanic whites	Referent		Referent		Referent		Referent		Referent	
Hispanics	0.916	*P* > 0.05	0.966	*P* > 0.05	1.059	*P* > 0.05	1.019	*P* > 0.05	1.023	*P* > 0.05
Blacks	0.954	*P* > 0.05	1.043	*P* > 0.05	0.972	*P* > 0.05	1.026	*P* > 0.05	0.969	*P* > 0.05
Asians and Pacific Islander	0.775	*P* < 0.01	0.791	*P* < 0.01	0.939	*P* > 0.05	1.03	*P* > 0.05	0.858	*P* < 0.01
Radiation										
Radiation not received	Referent		Referent		Referent		Referent		Referent	
Radiation received	0.693	*P* < 0.01	0.623	*P* < 0.01	0.586	*P* < 0.01	0.651	*P* < 0.01	0.917	*P* < 0.01
Cancer directed surgery										
Not performed	Referent		Referent		Referent		Referent		Referent	
Performed	0.231	*P* < 0.01	0.282	*P* < 0.01						

HR: hazard ratio; *P*: *P* value; Ca.: carcinoma.
